# A k-population model to calculate the firing rate of neuronal networks with degree correlations

**DOI:** 10.1186/1471-2202-15-S1-O14

**Published:** 2014-07-21

**Authors:** C Schmeltzer, A Kihara, I Sokolov, S Rüdiger

**Affiliations:** 1 Institut für Theoretische Physik, Humboldt Universität, Berlin, Germany, 12489, USA; 2 Universidade Federal do ABC, Santo André, Brazil

## 

Revealing the interplay of structure and function of the brain is one of the most intriguing topics in neuroscience.

**Figure 1:**
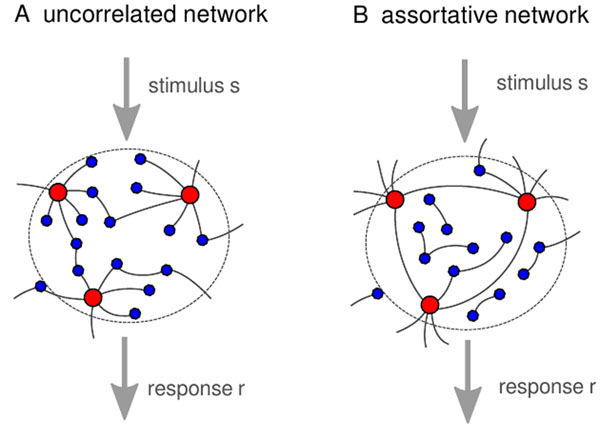
Schematic of the complex neural network. In the uncorrelated network (A), highly connected neurons (red dots) and poorly connected neurons (blue dots) are joined randomly. In the network with assortative degree correlations (B), neurons with similar connectivity are joined preferably. The network firing rate r is the response to a Poissonian external input current with rate s, which is injected into each neuron.

**Figure 2:**
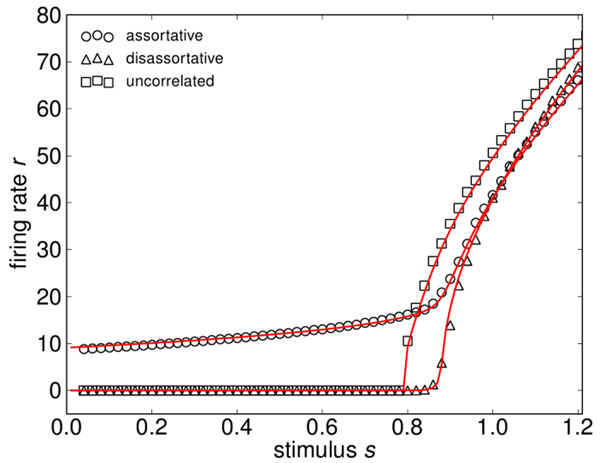
Firing rate of a heterogeneous network of integrate and fire neurons with in-degree correlations. Simulation results (dots) and theoretical predictions (lines). The assortative network shows sustained activity for very small stimuli.

The theory of complex networks is a promising approach to this aim, where one assumes that high cognitive processes arise as emergent properties of a network, in which many inane neurons are connected by a complex topology [1]. In this regard, we analyze analytically the emerging responses of networks with increasingly complex connectivity. We present a mathematical theory to calculate the firing rate of a network of leaky integrate-and-fire neurons, taking into account network features such as degree distributions and degree correlations (Figure 1). Heterogeneous connectivity and degree correlations have been shown to heavily influence network function and dynamics [2,3]. Our method is to divide the neuronal network in k-populations according to the number k of afferent synaptic links that connect to the neuron. Then, the steady state firing rates for these coupled populations can be calculated self-consistently. One of our main findings is that the population heterogeneity yields substantial deviations from mean-field calculations, where one ignores the network properties [4]. Importantly, our analysis shows that networks with assortative degree correlations lead to firing patterns even for sub-threshold inputs, where an uncorrelated network would not fire and thus, to a much larger sensitivity to low stimuli (Figure 2). Using information theory we further find an optimum in assortativity, with larger levels reducing again sensitivity for signal ensembles.
